# Chemistry

**Published:** 1876-10

**Authors:** 


Chemistry: General, Medical, and Pharmaceutical; including the Chemistry of the United States Pharmacopœia. A Manual of the General Principles of the Science, and their applications in Medicine and Pharmacy. By John Attfield, Ph. D., F.C.S., Professor of Practical Chemistry to the Pharmaceutical Society of Great Britain; formerly Demonstrator of Chemistry at St. Bartholomew's Hospital, London, etc. Seventh edition, revised from the sixth (English) edition, by the author. Philadelphia: Henry C. Lea. 1876.

The want of sufficient time to give this work a thorough perusal before the Journal must go to press, prevents an extended notice. From the scanning we have been able to give it, however, it seems to be admirably adapted to the use of medical students. It not only recognizes the new nomenclature of the American Pharmacopœia, but claims for its author the first proclamation of the principles upon which it is founded. The author also advocates the metric system of weights and measures proposed by the international conference, by which an easy and uniform system can be used in all nations.

